# Exploring Similarities Across the Space and Theater Industries

**DOI:** 10.3389/fpsyg.2020.574878

**Published:** 2021-01-22

**Authors:** Konstantin Chterev, Maria Eugenia Panero

**Affiliations:** ^1^Human Performance in Space, International Space University, Illkirch-Graffenstaden, France; ^2^School of Psychology, University of Surrey, Guildford, United Kingdom; ^3^Yale University, New Haven, CT, United States

**Keywords:** theater, space, astronauts, actors, theatre, creativity

## Introduction

“The earth is a very small stage in a vast cosmic arena” (Sagan and Druyan, 1997, p. 6).

This paper explores similarities across the space and theater industries. Both of these fields, for example, require people of the highest proficiencies to serve as the face of their project (i.e., astronauts and actors) while a mostly-unseen technical team supports the larger goal (i.e., mission control and backstage crews). We discuss a non-exhaustive list of domain-general factors (Plucker, 1998) while providing examples of domain-specific characteristics (Baer, 1998) encompassed within the expertise of astronauts and professional actors. While these domain-general factors may also be transferable across fields not covered here, we spotlight the space and theater industries as an illustrative example to further the call by research psychologists (e.g., de Vries, 2019) to investigate gaps within the literature of creativity in extreme environments.

## Creative Problem Solving

Solving problems on the job and in daily life requires creativity. When people need to solve work-related problems for which there is no precedence or pre-established procedure to follow, the creative employees are able to generate new and useful solutions. Creative problem solving can help societies cope with significant challenges in their environments (Sternberg and Lubart, 1996). Space and theater professionals are adept in creative problem solving to manage unplanned incidences successfully. As actors familiarize themselves with the intricacies of a script, astronauts learn the proper protocols to follow while working and experimenting in space modules. Although both astronauts and actors train for their performances for many months and years before their launch (Kanas, 2015), the ability to creatively resolve unexpected issues is beneficial to both domains.

Astronauts require a deep understanding of technical protocols to work with the equipment on which they rely. Nevertheless, Russian space psychologists recommend that creative abilities be considered one of the top traits when selecting and preparing future space travelers (Stepanova et al., 2003). Astronauts train in unscripted scenarios that test their ability to react to the unknown and they implement this knowledge in real missions (Kanas and Manzey, 2008). One example of an unpredictable problem solved successfully was illustrated during the 1966 Gemini VIII mission. Astronauts Neil Armstrong and David Scott were the first to link two spacecraft together in Earth orbit. Once they docked with the separately launched Agena, the two spacecraft began to spin quickly in the wrong direction. In an attempt to regain control, Scott switched the Agena off and on, which did not fix the problem. The astronauts therefore disengaged from the Agena, but the Gemini VIII continued to spin, which could have led to the astronauts losing consciousness. Before that happened, however, Armstrong regained control of the Gemini VIII by turning off the entire system and using the re-entry control system thrusters to stop the spin (Granath, 2016). Although this was not the kind of problem for which the crew had specific training or established procedure to follow, they were able to find a novel and useful solution to the life-threatening situation.

Actors learn to memorize huge amounts of text in short periods and recite it with spontaneity (Noice and Noice, 2006), but they also train to improvise their lines during misfiring props and mistimed cues that would otherwise threaten to ruin the illusion of the show (Gardner, 2013). The second author of this piece, who was an actor before becoming a research psychologist, offers a humorous personal anecdote of such an occurrence. In a run of a whodunit play, she portrayed the eccentric owner of an estate in which multiple murders were committed. During one night's performance, in the scene where she is to discover the murderer hiding in a secret passageway by sliding a bookcase panel (pulled open by a hidden crew member), the actor playing the murderer was distracted backstage and missed his cue. She was left momentarily standing alone in the middle of the stage with her arms open wide in the direction of the newly revealed opening to the secret passageway, pointing at nobody, while the audience waited. Without conscious awareness, she improvised the line, “I must be drinking too much again,” which fit perfectly with her character and situation, and was followed by raucous laughter from the audience. The collaboration between the swiftness of those backstage and the ability of actors to not panic can result in novel and useful solutions to prevent possible theatrical disasters because, as the phrase says, “The show must go on.”

## Personality Traits and Social Skills

Researchers have identified specific skills, as well as personality traits, associated with high performance in extreme environments, which are similar to those found in actors. The investment theory of creativity states that personality is a resource underlying creativity and that individuality and the willingness to tolerate ambiguity, to overcome obstacles and persevere, to grow (i.e., openness to experience), and to take sensible risks are related to creativity. Numerous studies have also identified intrinsic motivation and self-efficacy as personality attributes important in creative functioning (Sternberg and Lubart, 1991, 1996). Similarly, psychologists suggest that key personality traits for astronauts include strong achievement motivation, resiliency, adaptability, and high emotional stability. Using the “Big 5” personality test, space agencies look for astronaut candidates who score low in neuroticism and high in agreeableness, openness to experience, and conscientiousness (Rose et al., 1994; Kanas and Manzey, 2008; Kanas, 2015; Landon et al., 2018). With this same test, Nettle (2006) found that actors score higher than the general population in extraversion and openness to experience, as well as agreeableness, with a trend toward higher neuroticism. Furthermore, researchers have suggested that actors may be experts in emotion regulation (Ekman et al., 1983; Futterman et al., 1994; Pelletier et al., 2003; Gentzler et al., 2020). Within the framework of creativity theories, there may be opportunities for cross-industry research between the space and theater domains.

Both the astronaut and acting professions require social skills and competencies in communication, public speaking, and public relations. Within the space population, positive “instrumental” and “expressive” traits have been linked to relating well with others. Positive instrumental traits relate to high goal-orientation and need for achievement, whereas positive expressive traits relate to kindness and warmth (McFadden et al., 1994; Kanas and Manzey, 2008). Astronauts, like actors, often become ambassadors of their project, engaging with the public in myriad social events ranging from interviews to presentations. Thus, they require the ability to communicate effectively with a wide variety of audiences. One way to improve communication is by understanding the beliefs, intents, desires, and emotions of others, as well as being able to see through their perspective. Research shows that people with acting training have higher levels of theory of mind (the ability to infer the mental states of others) and empathy than those without this training (Nettle, 2006; Goldstein and Winner, 2010; Goldstein et al., 2013; Panero and Winner, 2020, in press). Therefore, part of the astronaut selection process and training revolves around endorsing specific social skills and personality traits that are often present in actors.

## Isolation

The skills and traits described above are not only imperative for interacting with audiences, but also within the isolated environments of the space and theater industries. Space missions and theater runs may involve many months in which astronauts and actors are away from their loved ones. Although rotations are common in these fields to help manage these challenges (e.g., backup astronauts and understudies), both careers often require casts and crews be separated from their homes. As we write this article, millions of people are self-isolating or quarantining to keep safe during the COVID-19 world pandemic. The mental health consequences emerging from these prolonged periods of social isolation include anxiety, obsessive-compulsive symptoms, post-traumatic stress, and depression (Pietrabissa and Simpson, 2020). Depression affects one's ability to function effectively at work by reducing the ability to solve problems. Social isolation has also been linked to reduced physical health and cognitive impairments, which would be particularly dangerous in life-threatening situations like the one described above faced by the Gemini VIII crew.

Astronauts must travel away from their families to live near their training facilities and, once the training is complete, eventually venture into outer space, farther away from those nearest to their hearts. Instead, they spend long periods with small work teams participating in activities ranging from high-stakes research experiments to monotonous chores. Space is the ultimate isolated, confined, and extreme environment; spending large amounts of time with people in a confined area may cause psychological changes, including impaired cognitive ability and interpersonal conflict. Awareness of these issues and effective communication may ease such tensions (Gushin et al., 1993; Palinkas and Suedfeld, 2008; Kanas, 2015; Landon et al., 2018; Pietrabissa and Simpson, 2020). Therefore, the National Aeronautics and Space Administration (NASA) provides “space flight resource management” trainings in which astronauts are taught effective crew coordination and team building, as well as strategies and support for psychological issues that may arise during the mission (Rogers et al., 2002; Palinkas and Suedfeld, 2008; Kanas, 2015).

While astronauts in orbit experience a different form of isolation than people on Earth, commonalities have been found between astronauts and people working abroad extensively (e.g., actors), including limited interaction with others and isolation from family and friends (Harris, 2009). Actors and other theater professionals often travel away from their hometown to pursue training or audition opportunities. Once they are cast in a show, they may move to yet another location to endure weeks of grueling daily rehearsals. Then, they may go on tour for an additional few months, performing at night and traveling to different locations during the day. Although research on actors, in particular, is lacking, research on the consequences of social isolation (e.g., Pietrabissa and Simpson, 2020) imply that these kinds of high-performance demands away from familial support may result in feelings of detachment or depression. Cross-industry research on this topic may explore the effects of and countermeasures for such issues that, for example, could inform future commercial spaceflight planned by organizations like SpaceX that recently ferried astronauts from NASA to the International Space Station and is paving the way in accessing missions to the Moon, Mars, and beyond.

## Pretend and Simulated Environments

Both astronauts and actors hone their skills by pretending to be somewhere else. During rehearsals and performances, stage sets are sometimes intentionally abstract or surprisingly intricate, replete with sliding panels and secret passageways, like those in a typical whodunit. Therefore, actors learn to engage their imagination and encapsulate themselves in the details of their surroundings—from the period and style of the furnishings, to the time of day and the temperature of the air. This allows them to establish a relationship with their scripted environment, which informs their performance. Characters (like people) behave differently when they are in a modern and familiar cozy cottage, for example, than when they are in a 1940s estate with a murderer on the loose. Furthermore, actors create a “fourth wall,” an imaginary barrier in the proscenium of the stage separating the actors from the audience, to remain enveloped within the imaginary location and circumstances of the play (Wilson and Goldfarb, 2012).

Astronaut training sites vary largely, but usually include simulations in habitats (facilities designed to simulate the physical and psychological environments of space). Astronauts engage in analog missions, which are Earth-based field activities set in extreme environments, such as Antarctica, that are similar, or analogous, to space (National Aeronautics Space Administration, 2020). While it is difficult to truly replicate space, certain aspects can be mimicked. NASA uses a neutral buoyancy laboratory, for example, that simulates microgravity by placing astronauts underwater as they conduct routine equipment and experiment training (National Aeronautics Space Administration, 2006). Another underwater analog habitat, called NEEMO, simulates emergency scenarios through which to persevere (National Aeronautics Space Administration, 2019). Although analog missions do involve the possibility of harm, should astronauts fail within these simulated environments, rescue crews are readily available. Therefore, during these trainings, astronauts may call on their imaginations to feel the degree of pressure as when in the real dangers of space from which salvation is less possible (Mohanty et al., 2006).

## Conclusion

This paper listed domain-general factors (Plucker, 1998) of the space and theater industries, including creative problem solving, social skills, personality traits, isolation, and pretend and simulated environments. Within each of these factors, domain-specific characteristics (Baer, 1998) corresponding to the expertise of astronauts and professional actors were explored. There are many more examples that could have been included, such as comparing the role of creativity in cognitive reappraisal for tolerating real or perceived threats within each field (see Kangas Dwyer and Davidson, 2012). Furthermore, the domain-general factors mentioned here (and others not mentioned) may potentially be transferable to other domains. Nevertheless, our hope is that this discussion will spark future cross-industry research on creativity in extreme environments.

## Author Contributions

KC and MEP collaborated on the outline of the paper, multiple drafts, and final manuscript. Both authors contributed to the article and approved the submitted version.

## Conflict of Interest

The authors declare that the research was conducted in the absence of any commercial or financial relationships that could be construed as a potential conflict of interest. The handling editor declared a shared affiliation, though no other collaboration, with one of the authors, MEP.
